# Functional chloroplasts in metazoan cells - a unique evolutionary strategy in animal life

**DOI:** 10.1186/1742-9994-6-28

**Published:** 2009-12-01

**Authors:** Katharina Händeler, Yvonne P Grzymbowski, Patrick J Krug, Heike Wägele

**Affiliations:** 1 Zoologisches Forschungsmuseum Alexander Koenig, Adenauerallee 160, 53113 Bonn, Germany; 2 Department of Biological Sciences, California State University, Los Angeles, California, 90032-8201, USA

## Abstract

**Background:**

Among metazoans, retention of functional diet-derived chloroplasts (kleptoplasty) is known only from the sea slug taxon Sacoglossa (Gastropoda: Opisthobranchia). Intracellular maintenance of plastids in the slug's digestive epithelium has long attracted interest given its implications for understanding the evolution of endosymbiosis. However, photosynthetic ability varies widely among sacoglossans; some species have no plastid retention while others survive for months solely on photosynthesis. We present a molecular phylogenetic hypothesis for the Sacoglossa and a survey of kleptoplasty from representatives of all major clades. We sought to quantify variation in photosynthetic ability among lineages, identify phylogenetic origins of plastid retention, and assess whether kleptoplasty was a key character in the radiation of the Sacoglossa.

**Results:**

Three levels of photosynthetic activity were detected: (1) no functional retention; (2) short-term retention lasting about one week; and (3) long-term retention for over a month. Phylogenetic analysis of one nuclear and two mitochondrial loci revealed reciprocal monophyly of the shelled Oxynoacea and shell-less Plakobranchacea, the latter comprising a monophyletic Plakobranchoidea and paraphyletic Limapontioidea. Only species in the Plakobranchoidea expressed short- or long-term kleptoplasty, most belonging to a speciose clade of slugs bearing parapodia (lateral flaps covering the dorsum). Bayesian ancestral character state reconstructions indicated that functional short-term retention arose once in the last common ancestor of Plakobranchoidea, and independently evolved into long-term retention in four derived species.

**Conclusion:**

We propose a sequential progression from short- to long-term kleptoplasty, with different adaptations involved in each step. Short-term kleptoplasty likely arose as a deficiency in plastid digestion, yielding additional energy via the release of fixed carbon. Functional short-term retention was an apomorphy of the Plakobranchoidea, but the subsequent evolution of parapodia enabled slugs to protect kleptoplasts against high irradiance and further prolong plastid survival. We conclude that functional short-term retention was necessary but not sufficient for an adaptive radiation in the Plakobranchoidea, especially in the genus *Elysia *which comprises a third of all sacoglossan species. The adaptations necessary for long-term chloroplast survival arose independently in species feeding on different algal hosts, providing a valuable study system for examining the parallel evolution of this unique trophic strategy.

## Background

Coevolution of host organisms and their specialized consumers, such as small-bodied herbivores or parasites, has been a major driver of speciation in terrestrial ecosystems [1-5]. For instance, tolerance of plant chemistry has facilitated shifts among chemically similar hosts by phytophagous insect lineages; novel hosts then act as distinct selective environments, fuelling the rapid evolution of host races and adaptive diversification of insects [6-11]. Characters associated with host exploitation, such as feeding or detoxification of secondary metabolites, therefore warrant special attention as evolutionary innovations that contribute to biodiversity [12-15]. Such traits have rarely been studied in aquatic systems, however, where the role of ecological associations in speciation remains poorly understood [16-20].

A rare adaptation that rests somewhere between endosymbiosis and predation is the retention of functional chloroplasts within the bodies of herbivores. Termed kleptoplasty, this phenomenon is documented in several marine protists including foraminifers [21], dinoflagellates [22] and ciliates [23]. However, chloroplast retention in the Metazoa is known only in the gastropod taxon Sacoglossa, a group of herbivorous sea slugs that feed mainly on green algae in the Ulvophyceae *sensu *Floyd and O'Kelly [24-32]. Taylor [33] mentioned kleptoplasts in the digestive system of another metazoan species, the rotifer *Ascomorpha ecaudis*. Subsequent investigations identified these "organelles" as whole algal organisms, usually called zoochlorellae [34].

**Figure 1:**
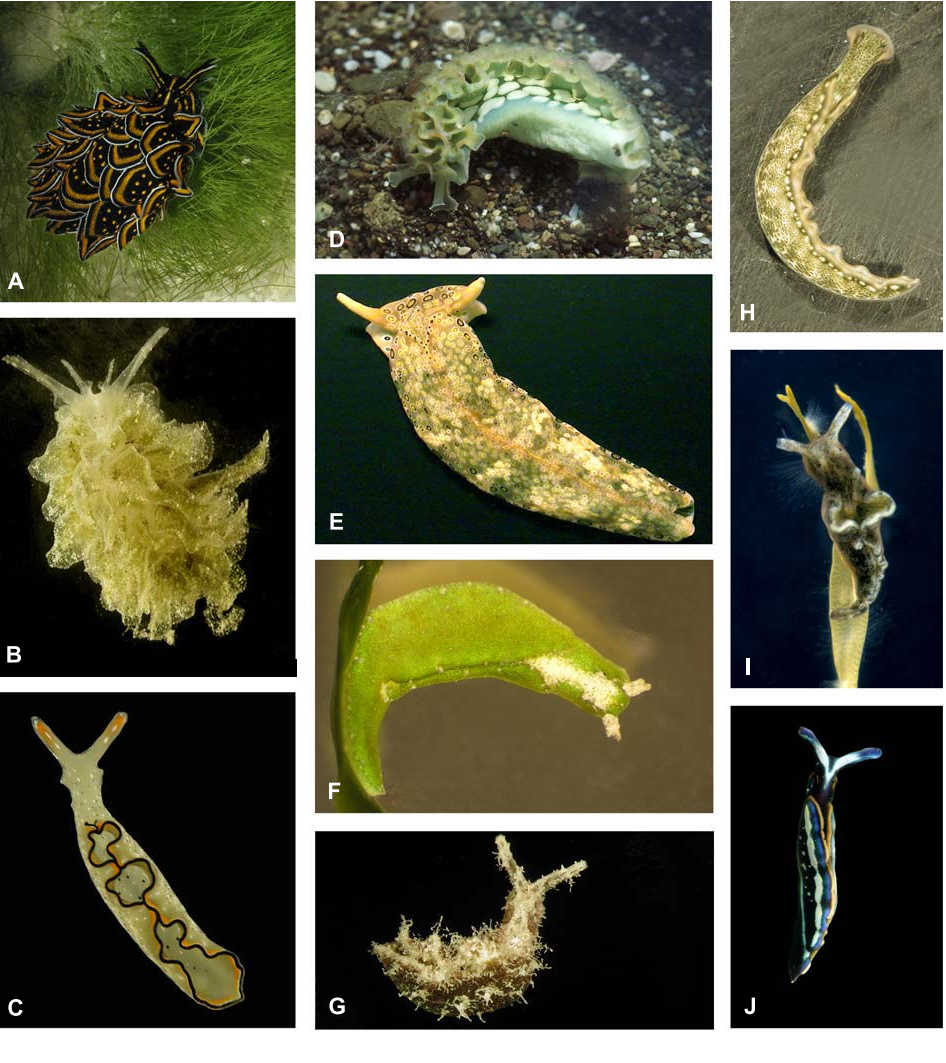
**Representative sacoglossans showing habitus typical of major plakobranchacean clades**. Approximation of length is given. (A) *Cyerce nigricans *on *Chlorodesmis fastigiata *(Lizard Island, Great Barrier Reef; 4 cm), (B) *Polybranchia orientalis *(Lizard Island, Great Barrier Reef; 2 cm), (C) *Elysia ornata *(Lizard Island, Great Barrier Reef; 2 cm), (D) *Elysia crispata *on sediment (Dominican Republic; 4 cm), (E) *Plakobranchus ocellatus *(Lizard Island, Great Barrier Reef; 4 cm), (F) *Elysia pusilla *(Maldives; 1 cm), (G) *Elysia tomentosa *(Lizard Island, Great Barrier Reef; 1.5 cm) (H) *Thuridilla carlsoni *(Lizard Island, Great Barrier Reef; 2 cm). (I) *Elysia viridis *(Mediterranean Sea, animal placed on brown algae; 1,5 cm). (J) *Thuridilla hopei *(Mediterranean Sea, 2 cm).

**Figure 2:**
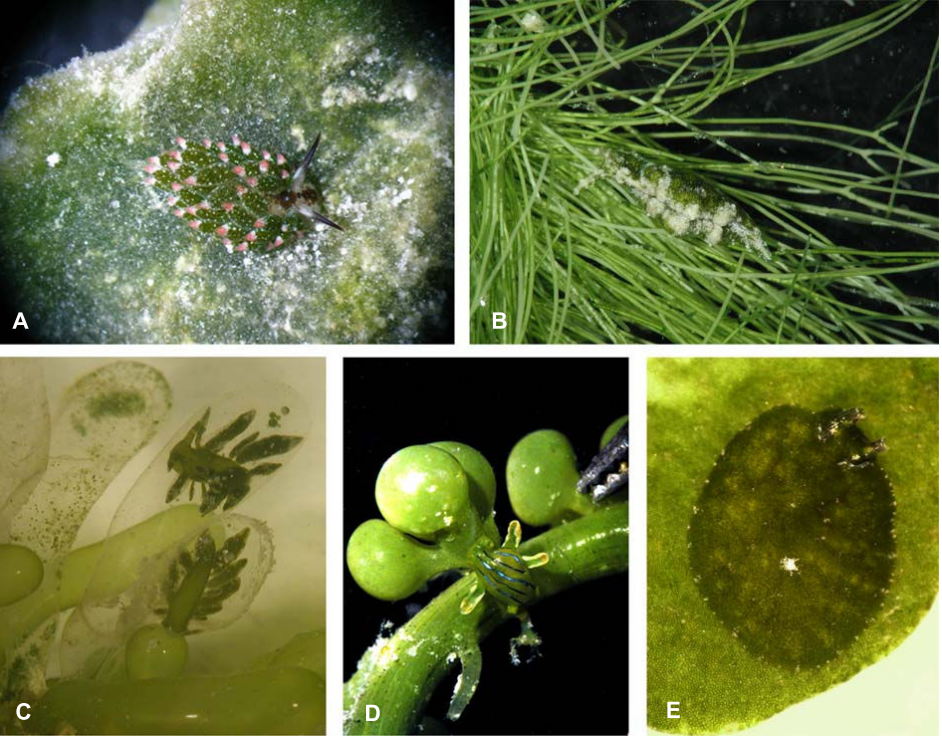
**Representative sacoglossans on their host algae**. Approximation of length is given. (A) *Costasiella *cf. *kuroshimae *on *Avrainvillea erecta *(Lizard Island, Great Barrier Reef; 1 cm), (B) *Elysia *spec. 5 found affiliated with *Chlorodesmis fastigiata *(Lizard Island, Great Barrier Reef; 1 cm), (C) *Ercolania kencolesi *inside of *Boergesenia *cf. *forbesii *(Lizard Island, Great Barrier Reef; 0.5 cm), (D) *Lobiger viridis *on *Caulerpa *spec. (Lizard Island, Great Barrier Reef; 0.5 cm), (E) *Bosellia mimetica *on *Halimeda tuna *(Mediterranean Sea; 1 cm).

Kleptoplasty may facilitate crypsis because plastids impart the host's color to the slug (Figures 1 and 2). However, the chief adaptive value is likely the nutritional benefit of photosynthetic assimilates released from chloroplasts into the slug cells [35-39]. This additional energy provided by the plastids may enhance slug survival during times of algal scarcity, when calcification impedes feeding, or when slugs migrate off of hosts in search of mates, new habitat patches or sites for oviposition [40,29,42].

Past studies indicated that sacoglossans vary widely in their capacity for photosynthesis [43,38]. However, the range of methods used to quantify plastid activity impedes generalization across studies. Using a Pulse Amplitude Modulated (PAM) Fluorometer, Wägele and Johnsen [44] demonstrated photosynthetic activity in *Plakobranchus ocellatus*, *Elysia expansa *and *Thuridilla gracilis *(as *T. ratna*), but detected no activity in *Cyerce nigricans *(Figure 1). Using the same methods over a longer time period, Evertsen et al. [45] demonstrated long-term retention of chloroplasts in *Plakobranchus ocellatus *and *E. timida*, whereas other *Elysia *spp. and *T. hopei *lost photosynthetic activity within a few days (Figures 1 and 2). The record for chloroplast retention is 14 months for *E. chlorotica *[46].

Wägele [47] postulated that functional kleptoplasty was a key character in the Sacoglossa, enhancing speciation by opening new adaptive zones. Although different efficiencies in chloroplasts' retention are known now for several decades, neither any attempt has been made to understand evolutionary history of this unique phenomenon within a metazoan group, nor the assumption of being a key character has ever been tested. We hypothesize that: 1. kleptoplasty is a key character promoting adaptive radiation in this group, 2. different stages in the evolution of kleptoplasty occurred and derived species are able to uphold photosynthetic activity better and longer, 3. a correlation of photosynthetic activity and sequestered food items exists. Testing these hypotheses requires a robust phylogeny. We therefore developed a phylogenetic hypothesis for the Sacoglossa based on molecular data.

We present data on photosynthetic rates for 29 species (186 specimens) representing all major groups within the Sacoglossa, including three of the four species known for high photosynthetic activity - *Plakobranchus ocellatus *(Indo-Pacific Ocean), *E. timida *(Mediterranean Sea) and *E. crispata *(Caribbean Sea) (Figure 1). Bayesian methods were used to infer phylogenetic relationships among extant taxa from nuclear and mitochondrial gene sequences, and to model the evolution of kleptoplasty based on new plastid retention data.

## Methods

### Molecular phylogeny of Sacoglossa

A molecular phylogenetic analysis of the Sacoglossa was undertaken to test conventional systematic groupings based on morphology (Table 1) [48], and to enable comparative analyses of trait evolution. *Cylindrobulla beauii *was designated as the outgroup for the analyses of Sacoglossa (see [26]).

**Table 1:** Sacoglossan systematics, based on cladistic analysis of morphological features [48].

Group names above family rank	Family	Genus
**Oxynoacea**	Volvatellidae Pilsbry, 1895	*Ascobulla *Marcus, 1972
		*Volvatella *Pease, 1860
	Juliidae E. A. Smith, 1885	*Julia *Gould, 1862
		*Berthelinia *Crosse, 1875
		*Tamanovalva *Kawaguti & Baba, 1959
	Oxynoidae G. & H. Nevill, 1869	*Oxynoe *Rafinesque, 1819
		*Roburnella *Marcus, 1982
		*Lobiger *Krohn, 1847

**Plakobranchacea**		
**Plakobranchoidea**	Plakobranchidae Gray, 1840	*Plakobranchus *van Hasselt, 1824
		*Thuridilla *Bergh, 1872
		*Elysia *Risso, 1818
		*Pattyclaya *Marcus, 1982
	Boselliidae Marcus,1982	*Bosellia *Trinchese, 1891
	Platyhedylidae Salvini-Plawen, 1973	*Platyhedyle *Salvini-Plawen, 1973
		*Gascoignella *Jensen, 1985
**Limapontioidea**	Polybranchiidae O'Donoghue, 1929	*Soghenia *Hamatani, 1991
		*Cyerce *Bergh, 1871
		*Mourgona *Marcus & Marcus, 1970
		*Polybranchia *Pease, 1860
		*Caliphylla *A. Costa, 1867
	Hermaeidae H. & A. Adams, 1854	*Hermaea *Lovén, 1844
		*Hermaeopsis *A. Costa, 1869
		*Aplysiopsis *Deshayes, 1839-53
	Limapontiidae Gray, 1847	*Placida *Trinchese, 1876
		*Stiliger *Ehrenberg, 1831
		*Alderia *Allman, 1846
		*Ercolania *Trinchese, 1872
		*Calliopaea *d'Orbigny, 1837
		*Olea *Agersborg, 1923
		*Limapontia *Johnston, 1836
	Costasiellidae Clark, 1984	*Costasiella *Pruvot-Fol, 1951

Sequences for a given species were generated in either Bonn, Germany or Los Angeles, California, U.S.A. For phylogenetic analysis, a concatenated dataset was generated from partial sequences of the mitochondrial 16S rDNA and *coxI *genes, and a partial sequence of the nuclear 28S rDNA gene. Loci were sequenced for at least two specimens of each species if specimens were available to confirm results for that taxon. In Bonn, some of the new 28S and *coxI *sequences were amplified from DNA extracts of the same specimens used previously by Händeler and Wägele [26] from which 16S sequences were obtained. All *coxI*, 16S and 28S sequences generated in Los Angeles were from a single specimen of each species. The final data set consisted of 72 sequences representing 63 nominal species (additional file 1).

DNA was extracted from alcohol-preserved specimens by means of NucleoSpin^® ^Tissue-Kit by Machery-Nagel or Dneasy^® ^Blood and Tissue Kit by Qiagen, following manufacturer's recommendations. Sequences were downloaded from Genbank or were generated using the following protocols and primer sequences given in Table 2.

**Table 2:** Primers used in PCR reactions.

gene	primer name	sequence 5' → 3'	origin
**16S**			
	16Sar-5'	CGCCTGTTTATCAAAAACAT	[101]
	16Sbr-3'	CCGGTCTGAACTCAGATCACGT	[101]
***coxI***			
	LCO1490	GGT CAA CAA ATC ATA AAG ATA TTG G	[60] after Folmer et al. 1994
	HCO2198	TAA ACT TCA GGG TGA CCA AAA AAT CA	[60] after Folmer et al. 1994
**28S**			
	28SC1	ACC CGC TGA ATT TAA GCA T	[102]
	28SD3	GAC GAT CGA TTT GCA CGT CA	[102]
	28SC2F(C2')*	GAA AAG AAC TTT GAA GAG AGA GT	[102]
	28SD2F	CCC GTC TTG AAA CAC GGA CCA AGG	[102]
	28SD2R	CCT TGG TCC GTG TTT CAA GAC GGG	[102]
	28SC2R(C2)*	ACT CTC TCT TCA AAG TTC TTT TC	[102]
	28SF2	GAGAGAGTTCAAGAGTACG	[103]
	28SR3	CTCAGGCATAGTTCACCATC	[103]
	28SF3	CGAAACCCAAAGGCGCAGTGA	[103]
	28SR1	AGCGCCATCCATTTTCAGGG	[103]

A portion of the *coxI *gene was amplified and sequenced in Bonn/Germany using primers LCO1490 and HCO2198. Amplification reactions (50 μl) consisted of 39.15 μl ddH2O, 5 μl 10× PCR Buffer without MgCl2 (Fermentas), 4 μl MgCl2 (25 mM), 0.15 μl Taq-Polymerase (5 U/μl), 0.4 μl of each Primer (10 pmol/μl) and 0.5 μl DNA. The PCR was carried out in the Gene Amp PCR System 9600 by Perkin Elmer^® ^under following conditions: 95°C for 240 s, followed by 9 Touch-down-cycles of 45 s at 94°C, 45 s at 56(-1)°C, 90 s at 72°C, followed by 25 amplification-cycles of 45 s at 94°C, 45 s at 48°C, 90 s at 72°C and a final extension at 72°C for 10 min followed by cooling down to 4°C. Amplicons were purified by means of NucleoSpin^® ^Extract II by Machery-Nagel, guided by the enclosed protocol. The mass of the amplicons was estimated by comparing ethidium bromide staining intensity of 5 μl of each purified reaction. Cycle sequencing reactions (Cycle Sequencing Kit BigDye^® ^Terminator v1.1 by Applied Biosystems) were carried out in the Gene Amp PCR System 9600 by Perkin Elmer^® ^under following conditions: 96°C for 120 s followed by 15 cycles of 10 s at 96°C, 5 s at 50°C, 150 s at 60°C followed by cooling down to 4°C.

Partial sequences of the nuclear 28S rDNA gene were produced in Bonn/Germany by amplifying with primers 28SC1 and 28SD3. Amplification reactions (50 μl) consisted of 39.15 μl ddH2O, 5 μl 10× PCR Buffer without MgCl2 (Fermentas), 4 μl MgCl2 (25 mM), 0.15 μl Taq-Polymerase (5 U/μl), 0.4 μl of each Primer (10 pmol/μl) and 0.5 μl DNA. The PCR was carried out in the Gene Amp PCR System 9600 by Perkin Elmer^® ^under following conditions: 95°C for 240 s, followed by 38 amplification-cycles of 30 s at 94°C, 30 s at 52.5°C, 150 s at 72°C and a final extension at 72°C for 10 min followed by cooling down to 4°C. Amplicons were purified by means of NucleoSpin^® ^Extract II by Machery-Nagel, guided by the enclosed protocol. The mass of the amplicons was estimated by comparing ethidium bromide staining intensity of 5 μl of each purified reaction. Cycle sequencing reactions (Cycle Sequencing Kit BigDye^® ^Terminator v1.1 by Applied Biosystems) were carried out in the Gene Amp PCR System 9600 by Perkin Elmer^® ^under following conditions: 96°C for 120 s followed by 15 cycles of 10 s at 96°C, 5 s at 50°C, 150 s at 60°C followed by cooling down to 4°C. Internal primers 28SC2F(C2')*, 28SD2F, 28SD2R and 28SC2R(C2)* were used if necessary to gain both strains. Reaction products were sequenced using an ABI PrismTM 377 DNA Sequencer by Applied Biosystems or with service of Macrogen inc, Korea.

Partial sequences of the *coxI *and 16S genes were produced in Los Angeles, California, U.S.A. as described in Ellingson and Krug [49]. Partial 28S sequences were generated as described in Krug et al. [50].

Sequences were aligned using the web server of MAFFT [51,52]. Because different primers were used by our respective labs to amplify 28S, sequences produced in Los Angeles were cut to end with sequences produced with primers 28S1 and 28SD3. The *coxI *data showed evidence of mutational saturation at the 3^rd ^codon position, thus only the 1^st ^and 2^nd ^codon positions were included in phylogenetic analysis. Final length of aligned and concatenated sequences was 2117 bp: 28S rDNA gene, positions 1-1220, 16S rDNA gene, positions 1221-1679, and *coxI *(1^st ^and 2^nd ^position), positions 1680-2117.

Phylogenetic analysis by Bayesian Inference was performed using Metropolis-Coupled Markov-Chain Monte Carlo methods as implemented in the program BayesPhylogenies [53]. This program uses a mixture model to accommodate among-site heterogeneity in patterns of sequence evolution. Parameters are estimated for a user-defined number of general time reversible (GTR) models of sequence evolution, and the most likely model is assigned to each position in the alignment. Mixture models should equal or out-perform analyses that assign one model to all sites in a given gene or codon partition. Our analyses used three GTR models, each estimating base composition separately and including a parameter for gamma-distributed rate heterogeneity (4 rate classes). Four chains (three heated and one cooled) were run for 2,65 million generations, sampling trees every 1000 iterations of the Markov chain. Based on inspection of log-likelihood scores, the first 1,650 trees were discarded as burn-in. From the retained tree sample, a 50% majority-rule consensus phylogram was constructed with mean branch lengths in the program BayesTrees, and posterior probabilities calculated to estimate nodal support.

### PAM measurements

The additional file 2 provides details of the specimens and species used in the present study for analysing retention of chloroplasts. Analyses of photosynthetic activity were performed with a Pulse Amplitude Modulated Fluorometer (DIVING PAM, Walz, Germany) by measuring the maximum quantum yield of chlorophyll a fluorescence in photosystem II usually within the first day after collection of animals from their food algae (with the exception of *Plakobranchus *and *Thuridilla *species, where food source is not known). The fiber optics from the PAM were usually placed five mm above the animal. Measurements were taken with chloroplasts acclimated to darkness, thus with all reaction centres open and a minimal fluorescence emission. While measuring, an actinic light probe is emitted which induces closure of reaction centres and yields maximum emission of fluorescence. For further details of the methods see Wägele and Johnsen [44] and Burghardt and Wägele [54].

In general three or more measurements per animal per day were taken at intervals of over 15 minutes to ensure regeneration of reaction centres and acclimation of chloroplasts to darkness again. Usually animals were kept in small aquaria with a running through water system (25°C to 28°C) or water was changed on a daily base. They were exposed to natural day-night rhythm, but not directly to the sun. Shading was given by plastic roofs or plastic sheaths, which reduced irradiance of the sun (up to 400 μmol quanta per square meter and second), but did not filter certain spectra of the light [pers. comm. Eva McClure, Brisbane]. Animals kept in closed rooms were exposed to sunlight through windows, or light-dark rhythms were imitated by artificial light sources.

When multiple specimens were available, all specimens were measured for initial documentation on photosynthesis, but on long term, only the bigger animals were chosen for analyses, since body sizes below 5 mm yield less reliable measurements. Mean values were calculated from daily measurements taken for each specimen. These values (for individuals) were then pooled to yield a mean and standard deviation for each species (additional file 3). Best-fit trendlines were calculated with Microsoft Office Excel to examine changes in photosynthetic activity over time. For some species only an initial yield value was obtained because insufficient specimens were available for extended measurements.

### Reconstruction of ancestral character state

Ancestral character state reconstruction was used to infer when short-term plastid retention evolved in the Sacoglossa, and how many independent origins were likely. Pagel et al. [55] provide a Bayesian method for estimating ancestral states of discrete traits [56], combining the probability that a node exists with the probability of a particular state at that node. Continuous-time Markov models of trait evolution were fit to the data with the program *BayesMultistate *using the MCMC sample of phylogenetic trees generated as described above. This method incorporates phylogenetic uncertainty, as the frequency of a given node in the tree sample limits the statistical confidence in character state reconstructions for that hypothetical ancestor [57,55]. Each taxon for which PAM data were available was coded as having one of three character states: non-functional chloroplast retention, short-term retention (inability to digest chloroplasts), and long-term retention. Other taxa were coded as missing data if PAM measurements were not available.

A Markov chain was used to estimate posterior probabilities of distributions of the six transition rates between the three alternative character states, and to estimate the ancestral state at each node. A reversible-jump model was used to obtain priors, with an exponential prior seeded from a uniform hyperprior on the interval 0 to 30. The ratedev parameter, which determines the magnitude of changes proposed to rate coefficients, was set to 20 based on pilot runs. These values gave acceptance rates for model parameters of about 20%. After a burn-in period of 10^5 ^iterations, chains were sampled for rate coefficients and their associated trees (every 500 generations) to yield 40,000 sampled parameter sets. The posterior probability distribution of character states was estimated for the node representing the last common ancestor of the Plakobranchoidea using the "most recent common ancestor" (MRCA) technique in *BayesMultistate*. The character state of this node was then fixed as each of the possible states in separate runs using the "fossil" command, and a Markov chain run for 20 million generations as described above, sampling every 500 generations after the burn-in period. These procedures were repeated for the last common ancestor of *Elysia chlorotica *and *E. crispata*, to determine if these species represent independent origins of long-term retention or if there was one origin in the ancestor of the clade to which both *Elysia *spp. belong.

The log of the harmonic mean of the likelihood scores for successive iterations of the chain was calculated for each model. Log-Bayes factor (BF) tests were then used to compare the likelihood of models where the ancestral state was fixed as short-term retention against models where the ancestral state was fixed as non-retention or long-term retention. The BF test statistic is twice the difference in the log-likelihood scores of two models; BF values from 2-5 are taken as positive evidence, while a BF >5 is strong evidence favoring the more likely model [58].

## Results

### Molecular phylogeny of Sacoglossa

**Figure 3:**
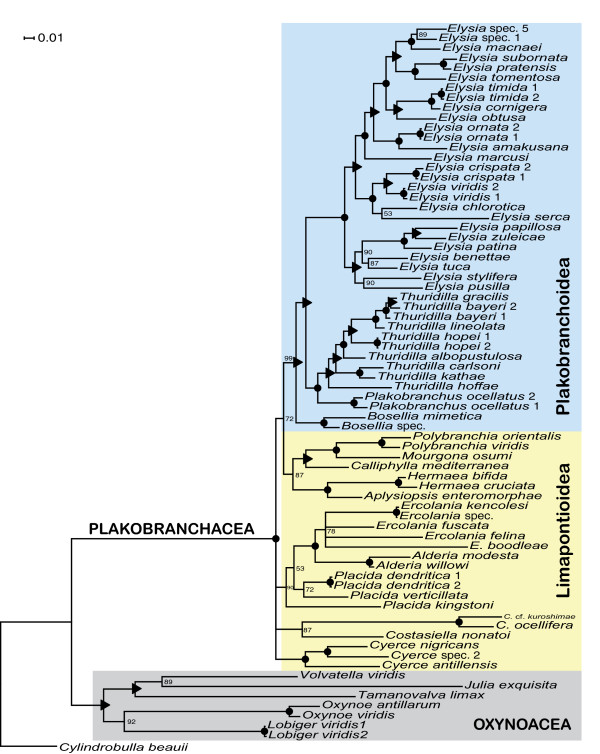
**Phylogeny of Sacoglossa**. A Bayesian analysis was performed on concatenated partial gene sequences of the nuclear 28S, the mitochondrial 16S, and the mitochondrial *coxI *(1^st ^and 2^nd ^positions only) loci. Shown is a 50% majority-rule consensus tree generated from the post-burn-in tree sample. Numbers above branches are posterior probability support, black dot: posterior probability = 100, black triangle: posterior probability = 95 - 99.

Phylogenetic analysis of three gene regions strongly support monophyly for the two major sacoglossan lineages: the Oxynoacea, comprising all shelled Sacoglossa, and the shell-less Plakobranchacea, comprising Plakobranchoidea and Limapontioidea (Figure 3). Within the Plakobranchacea, the group Plakobranchoidea is well resolved and strongly supported with a posterior probability (PP) of 0.99. The monotypic family Boselliidae, containing only the genus *Bosellia *(Figure 2E), was sister taxon to the well-supported family Plakobranchidae which includes the species with parapodia, lateral flaps of the body wall that cover the dorsum. Each of the three genera in the Plakobranchidae (*Elysia*, *Thuridilla *and *Plakobranchus*) was monophyletic.

Relationships of the main subgroups of the Limapontioidea were not resolved. Limapontiidae (PP = 0.90) and the monotypic Costasiellidae (PP = 0.87) were monophyletic (Figure 3). Only one traditional family, the Polybranchiidae, was not monophyletic in our analysis, due to the exclusion of the genus *Cyerce*. The sister taxon of this "reduced" family was Hermaeidae (*Hermaea *+ *Aplysiopsis*). The resulting monophylum was sister taxon to the Plakobranchoidea (PP = 0.72).

### PAM measurements

Comparing all available measurements of yield values throughout the Sacoglossa, three distinct levels of photosynthetic activity were distinguished: 1. No functional retention, with disintegration of chloroplasts within few hours, resulting in yield values lower than 0.2 on the very first day of collecting. 2. Short-term functional retention, with initial yield values above 0.5. These values last for one or two days, and then drop within seven to 14 days. 3. Long-term functional retention, with yield values of more than 0.4 even after 20 days.

**Figure 4:**
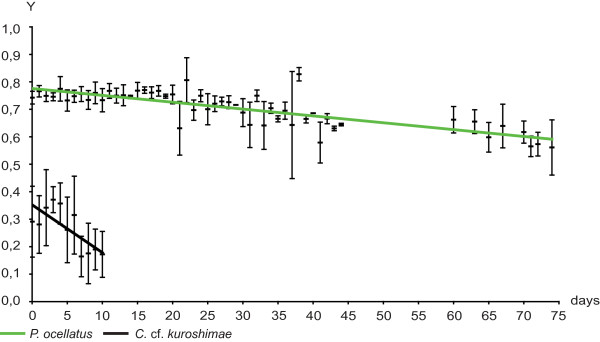
**Yield values (PAM measurements) of *Costasiella *cf. *kuroshimae *compared to *Plakobranchus ocellatus***. Y is the yield value of chloroplasts' photosynthesis that has been measured every day after capture of animal. Y is plotted against the days of starvation. Trendlines are calculated in Excel. In every figure the species with the longest measured ability of chloroplasts' function, *Plakobranchus ocellatus*, is shown as reference. Black trendline indicates no functional retention. Trendlines of species with long-term retention are green. Standard deviation and mean values are given.

Members of the shelled group Oxynoacea exhibited no yield values (Table 3), although ground fluorescence was high in some cases. High ground fluorescence with no yield indicates the presence of chlorophyll a, but a loss of plastid functionality. No species of all four sampled limapontioidean families exhibited photosynthetic activity, indicating no functional retention of chloroplasts in Limapontioidea. One potential exception was *Costasiella *cf. *kuroshimae *which had a starting yield value of 0.35, decreasing to < 0.2 within eleven days (Figure 4). Yield values, standard deviation and a corresponding trendline (black line) is shown in comparison to yield values, standard deviation and trendline of *Plakobranchus ocellatus *(green line) representing the slug with the highest and longest photosynthetic activity in this study.

**Table 3:** Duration of chloroplast functionality in representative sacoglossans, based on PAM measurements of photosynthetic yield.

species	initial yield	days	final yield	retention
Oxynoacea				
*Oxynoe antillarum*	0	-	0	non-functional
*Oxynoe *cf. *viridis*	0	-	0	non-functional
*Lobiger viridis*	0	-	0	non-functional
*Julia exquisita*	0	-	0	non-functional
*Julia *spec.	0	-	0	non-functional
Plakobranchacea				
Limapontioidea				
*Costasiella *cf. *kuroshimae*	0.35	11	0.18	non-functional
*Costasiella nonatoi*	0	-	0	non-functional
*Cyerce antillensis*	0	-	0	non-functional
*Cyerce nigricans*	0	-	0	non-functional
*Ercolania kencolesi*	0	-	0	non-functional
*Placida dendritica*	0	-	0	non-functional
*Polybranchia orientalis*	0	-	0	non-functional
Plakobranchoidea				
*Bosellia mimetica*	0.56	9	0.06	short-term
*Elysia bennettae*	0.60	9	0.5	short-term
*Elysia cornigera*	0.70	11	0.33	short-term
*Elysia crispata*	0.65	40	0.26	long-term
*Elysia ornata*	0.68	13	0.15	short-term
*Elysia pusilla*	0.53	15	0.15	short-term
*Elysia subornata*	0.24	8	0.14	non-functional
*Elysia timida*	0.70	35	0.53	long-term
*Elysia tomentosa*	**0.57**	-	-	short-term
*Elysia viridis*	**> 0.56**	-	-	short-term
*Elysia zuleicae*	**0.38**	-	-	non-functional
*Elysia *spec. 1	0.61	9	0.40	short-term
*Plakobranchus ocellatus*	0.78	74	0.59	long-term
*Thuridilla carlsoni*	0.70	7	0.26	short-term
*Thuridilla gracilis*	0.51	12	0.20	short-term
*Thuridilla hopei*	0.40	8	0.11	short-term
*Thuridilla kathae*	**0.59**	-	-	short-term

Initial and final yield values were extrapolated with help of Excel. Initial yields of species for which only start yield was available are bolded. First authors of species descriptions see additional file 1.

**Figure 5:**
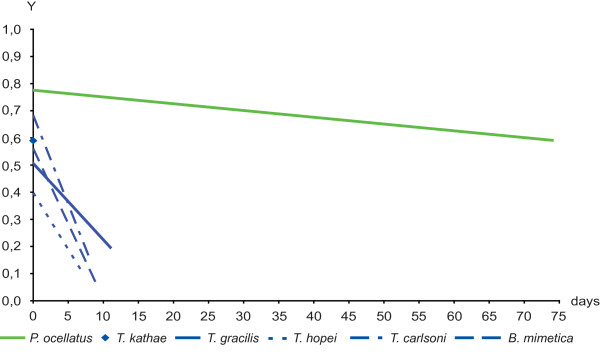
**Yield values (PAM measurements) of the genera *Bosellia *and *Thuridilla *compared to *Plakobranchus ocellatus***. Y is the yield value of chloroplasts' photosynthesis that has been measured every day after capture of animal. Y is plotted against the days of starvation. Trendlines are calculated in Excel. In every figure the species with the longest measured ability of chloroplasts' function, *Plakobranchus ocellatus*, is shown as reference. Trendlines of species that had short-term retention are blue. Trendlines of species with long-term retention are green. For better clarity standard deviation and mean values are not included.

**Figure 6:**
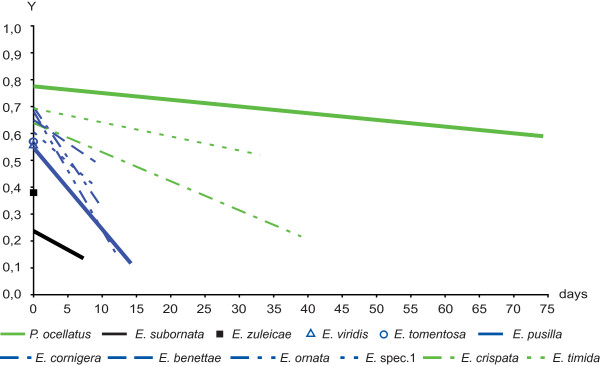
**Yield values (PAM measurements) of the genus *Elysia *compared to *Plakobranchus ocellatus***. Y is the yield value of chloroplasts' photosynthesis that has been measured every day after capture of animal. Y is plotted against the days of starvation. Trendlines are calculated in Excel. In every figure the species with the longest measured ability of chloroplasts' function, *Plakobranchus ocellatus*, is shown as reference. Black trendlines indicate no functional retention. Trendlines of species that had short-term retention are blue. Trendlines of species with long-term retention are green. For better clarity standard deviation and mean values are not included.

In contrast, all members of Plakobranchoidea had initial yield values from 0.5 to 0.8 (Figure 5 and 6). Members of the genus *Thuridilla *had short-term retention with initial yield values above 0.5, but their yield decreased to ~0.2 within a few days. Two specimens of *T. kathae *had equivalent initial yield values but decline in yield varied markedly between individuals (additional file 3). The lowest initial yield value was obtained for *T. hopei *(0.4), but its decline was similar to that of the other *Thuridilla *spp. *Bosellia mimetica *had short-term retention, initial yield value was 0.56, but yield decreased to less than 0.1 within nine days. Figure 5 compares the trendlines of the species of the genus *Thuridilla *and *Bosellia mimetica *(blue lines) with *Plakobranchus ocellatus *(green line) as reference. Details are listed in the additional file 3. Species of *Elysia *varied greatly in their photosynthetic ability. Many species (*E. bennettae*, *E. ornata, E. pusilla, E. cornigera, E. tomentosa*, *E. viridis *and *Elysia *spec. 1) started with yield values of 0.5-0.7 immediately after collection, but yields decreased to <0.4 within one to two weeks. In contrast to most *Elysia *spp., *E. subornata *and *E. zuleicae *exhibited very low values directly after collection - 0.24, and 0.38, respectively.

Three species - *E. crispata*, *E. timida *and *Plakobranchus ocellatus *- exhibited greatly prolonged retention of chloroplast activity (Figure 6). Significant yield values of *E. crispata *persisted for 40 days, at which point they had dropped to 0.22. Yield values of *E. timida *remained >0.52 after 35 days. Maximum retention was measured in *P. ocellatus*, which had a mean initial value 0.78 decreasing to 0.59 over 74 days. Figure 6 compares trendlines of members of *Elysia *with *P. ocellatus *as reference. Trendline of *E. subornata *and initial yield value of *E. zuleicae *are black indicating no functional retention for the measured specimens. Trendlines of species that had short-term retention (*E. bennettae, E. ornata, E. pusilla, E. cornigera, E. tomentosa*, *E. viridis *and *Elysia *spec. 1) are blue. Trendlines of *E. crispata*, *E. timida *and *P. ocellatus *with long-term retention are green. Details are listed in the additional file 3.

**Figure 7:**
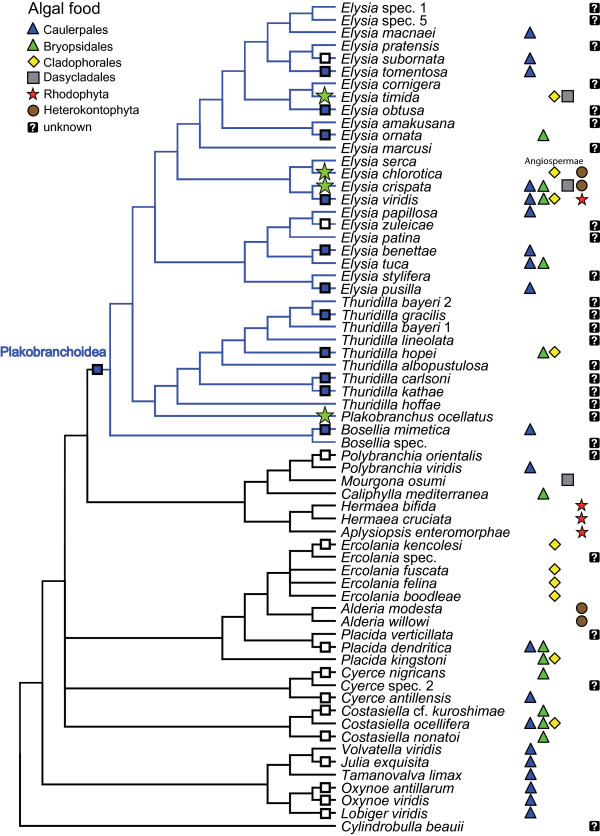
**Evolutionary patterns in photosynthetic activity and host use in the Sacoglossa**. Information on host algae was taken from Händeler & Wägele [26], Händeler et al. (unpublished data), Teugels et al. [98], Trowbridge [99]; Krug et al. [100] and authors' data. Empty square: no-functional chloroplast retention, blue square: functional short-term chloroplast retention, green star: functional long-term chloroplast retention.

The photosynthetic activity for each species was classified as either no functional retention, short-term retention or long-term retention. Character states were mapped on a simplified cladogram (Figure 7).

### Reconstruction of ancestral character state

For character state reconstruction, taxa were grouped into three categories of photosynthetic activity as outlined above (Figure 7). For a few species like *Thuridilla kathae *and *Elysia tomentosa*, only initial yield values were available but these were comparable to values for species that maintained photosynthetic ability for about a week; we therefore scored such species as having short-term retention, pending additional study.

Ancestral character state reconstructions statistically supported one origin of short-term chloroplast retention, in the most recent common ancestor (MRCA) of the Plakobranchoidea (Table 4; Figure 7). The posterior probability that the MRCA had short-term retention was estimated as 89.1% ± 18.0% SD. Models of trait evolution in which the MRCA was fixed as having short-term retention were strongly favoured over models in which the MRCA had no retention (BF test = 7.31), and positively supported over models in which the MRCA had long-term retention (BF = 3.92). There were two independent losses of short-term retention (*Elysia zuleicae*, *E. subornata*) (Figure 7).

**Table 4:** Reconstructed ancestral character states for chloroplast retention in the most recent common ancestor of (A) the Plakobranchoidea, and (B) the clade comprising *E. chlorotica*, *E. crispata*, *E. serca *and *E. viridis*.

Clade	Character state	Posterior probability	log(harmonic mean of likelihoods)	BF test statistic 2 [logA-logB]
A	non-retention	0.005 ± 0.016	-31.9672	7.31
	short-term retention	0.891 ± 0.180	-28.3106	-
	long-term retention	0.103 ± 0.179	-30.2700	3.92

B	non-retention	0.002 ± 0.011	-35.4964	14.43
	short-term retention	0.526 ± 0.261	-28.2813	-
	long-term retention	0.472 ± 0.261	-30.4022	4.24

Taxa were coded as having non-retention, short-term retention, or long-term retention of functional chloroplasts based on PAM data. The evolution of kleptoplasty was then modeled using Bayesian Inference via Markov-Chain Monte Carlo methods to estimate the posterior probability of alternative character states. Posterior probabilities (means ± one standard deviation) were calculated from an MCMC chain run for 20 million generations. Bayes factor (BF) tests were used to compare log-likelihood scores of models in which the ancestor was assigned one of the possible character states; values >2 represent positive evidence and values >5 represent strong evidence for models fixing the ancestral state as short-term retention.

The closely related *Elysia chlorotica *and *E. crispata *both express long-term chloroplast retention, but feed on very different host algae. We therefore tested whether long-term kleptoplasty was present in their MRCA or evolved independently in each species. The posterior probability of short-term chloroplast retention was slightly higher than that of long-term retention for the MRCA of *E. chlorotica *and *E. crispata*, and models in which their MRCA was fixed as having short-term retention were supported over models in which their ancestor had long-term retention (BF test = 4.24) (Table 4). This result supports independent origins of long-term functionality in *E. crispata *and *E. chlorotica*.

## Discussion

### Molecular phylogeny of Sacoglossa

Bayesian analysis of three gene sequence regions revealed two monophyletic groups within the Sacoglossa, Oxynoacea and Plakobranchacea, confirming the morphological analysis by Jensen [48]. In the present study, improved taxon sampling and addition of nuclear gene sequence data led to the recovery of a monophyletic Oxynoacea, in contrast to prior molecular analyses based on fewer species and mtDNA alone [26]. Within the Plakobranchacea, Plakobranchoidea was strongly supported as monophyletic, with the monotypic family Boselliidae as sister taxon to the Plakobranchidae. Contrary to Jensen [48], but concordant with Gosliner [59] and Bass and Karl [60], *Plakobranchus *and *Thuridilla *were sister taxa.

Monophyly of the cerata-bearing sacoglossans, usually united as Limapontioidea, was not supported in our study, instead major limapontioidean groups formed a polytomy; improved taxon sampling and sequence data from additional loci may solve this problem. The traditional inclusion of *Cyerce *in the Polybranchiidae was challenged by our finding of a well supported sister taxon relationship between Hermaeidae and part of the Polybranchiidae (*Polybranchia *+*Caliphylla *+*Mourgona*); this merits further attention. The traditional Hermaeidae (*Hermaea *and *Aplysiopsis*) was recovered. *Costasiella *did not show affinity for the other genera classified in the Limapontiidae (*Alderia, Ercolania *and *Placida*), but has also been previously placed in its own family.

Our phylogenetic results indicate that the Caribbean "*Bosellia marcusi" *described by Marcus [61] is a derived species of *Elysia*. Morphological examination indicates that the parapodia of "*B. marcusi*" have secondarily fused over the dorsum, producing a superficial similarity with *Bosellia *[P. Krug, unpublished data].

### Evolution of functional chloroplast retention

Based on our phylogeny and character state reconstructions, there was one probable origin of short-term chloroplast retention in the last common ancestor of the Plakobranchoidea, and four independent origins of long-term retention. No species in the Oxynoacea and Limapontioidea were able to maintain photosynthetic activity, based on PAM measurements. Functional chloroplast retention was not detected in five oxynoacean species representing the basal shelled sacoglossans (Table 3). In species with no functional retention, chloroplasts are phagocytosed by digestive glandular cells and rapidly disintegrate [62,63]. Clark and Busacca [64] concluded that *Oxynoe *retains chloroplasts because they were able to isolate chlorophyll from slugs, but they did not detect net fixation of CO_2_. We measured high ground fluorescence in oxynoaceans but very low yield values, indicating free chlorophyll but no functional chloroplasts.

Similarly, all limapontioidean species except *Costasiella *cf. *kuroshimae *had yield values <0.2, indicating no chloroplast retention. This confirms studies on *Placida dendritica *[62,63] and *Aplysiopsis enteromorphae *(= *Hermaeina smithi*) [65]. We detected initial yield values of 0.3-0.4 for *C*. cf. *kuroshimae *6-8 hr after collection, whereas the host alga *Avrainvillea *gave yield values >0.6, leading to the conclusion that chloroplasts are quickly degraded in *C*. cf. *kuroshimae*. A former study based, amongst others, on ultrastructural analyses reported intact chloroplasts and carbon-fixation for about 10 days in a specimen of the congener *C. ocellifera *starved for two weeks [66], so additional PAM studies on *Costasiella *spp. are warranted.

No specimens of *Hermaea*, a genus of red algae feeders, were available for PAM measurements. Short-term retention of functional chloroplasts from the rhodophyte *Griffithsia flosculosa *was reported for *H. bifida*, based on O_2 _production and ^14^C incorporation in slug tissue [67,68], but experimental methods were not adequately described [67]. Further studies are therefore needed to assess the fate of chloroplasts within Limapontioidea.

Short- and long-term functional retention were only found in the Plakobranchoidea. Chloroplast function was not detected in *Elysia subornata *and *E. zuleicae *from the Caribbean. Based on ancestral character state reconstruction, these lineages represent independent losses of short-term retention, which arose once in the plakobranchoidean ancestor. However, an absence of functional chloroplasts may result from several causes (see discussion of method below), therefore these species should be reinvestigated. Long-term retention was documented for *E. crispata*, *E. timida *and *Plakobranchus ocellatus*. Our study shows for the first time that functional retention in *E. crispata *lasts up to 40 days, expanding prior studies based on oxygen production [69,70,37]. We confirmed stable long-term retention in *E. timida*, also described by Evertsen et al. [45]. Kleptoplasty may be an ecologically necessity for *E. timida *as an energy source when algal hosts are heavily calcified or in short supply [40]. The highest rates of photosynthesis occurred in *P. ocellatus*, lasting over a month with remarkable stability. This is a large species by sacoglossan standards (up to five cm long), and the subject of prior studies on chloroplast retention [[71], as *P. ianthobapsus*, [72], as *P. ianthobapsus*, [27,45]]. *E. chlorotica*, a species best known for its long-term retention, was not available for PAM measurements but its photosynthetic ability is well documented [46,73-77].

Given that short-term retention was an apomorphy of the Plakobranchoidea, there were likely at least four independent gains of long-term function in the derived species noted above. Ancestral character state reconstructions returned mild support for two independent origins in *E. chlorotica *and *E. crispata*. The related species *E. viridis *feeds on six genera of host algae [C. Trowbridge, pers. comm.] and may be capable of long-term chloroplast retention from a subset of those hosts, which warrants future study. However, when ancestral character state analyses were rerun coding *E. viridis *as having long-term chloroplast functionality, the results were unchanged with respect to independent origins in *E. chlorotica *and *E. crispata*.

We propose that functional chloroplast retention evolved in two sequential steps. The first step was *loss *of the ability to rapidly digest dietary plastids after phagocytosis by digestive cells. A pre-adaptation for prolonged chloroplast retention, this step produces short-term functionality because undigested chloroplasts perform photosynthesis without support from the (digested) algal nucleus, for as long as the chloroplasts survive. Many sacoglossan host algae are large-celled and coenocytic, including numerous Chlorophyta and the heterokontophyte *Vaucheria*. Coenocytic algae may require resilient chloroplasts for cytoplasmic streaming or thallus wound-plug formation (giant-celled and coenocytic Chlorophyta: [78], *Vaucheria*: [79]). The toughness of chloroplasts may in turn have facilitated the evolutionary transition to short-term retention in the ancestral plakobranchoidean.

The second requirement for long-term retention is to prolong chloroplast survival by supplying essential proteins such as components of the light-harvesting complex, enzymes involved in chlorophyll biosynthesis, etc. The encoding genes are normally found in the algal nucleus, but in at least *Elysia chlorotica *have entered the slug's genome by horizontal gene transfer [80,81]. This strategy is distinct from the reliance on transcriptionally active nuclei, as occurs in ciliates that incorporate plastids from cryptophytes [23]. Future research should determine whether horizontal gene transfer happened once in the plakobranchoidean ancestor, or if gene transfer happened independently in each lineage capable of long-term retention. If gene transfer happened early, then the relevant genes were transcriptionally activated or "switched on" independently in each of the four species capable of prolonged photosynthetic activity. Genetic analysis of selected sacoglossan taxa are planned to resolve this issue, which has fundamental implications for understanding endosymbiosis and the evolution of chimeric genomes [82].

A second question is whether short- versus and long-term retention is dependant on the type of chloroplasts in the host algae or on some properties of the slugs' digestive cells that affect how the cells deal with sequestered chloroplasts. To explore possible correlations between mode of retention and algal type, available data on host use were mapped onto the cladogram (Figure 7). All members of the Oxynoacea feed on the genus *Caulerpa *(Caulerpales) and seem to digest them directly. Several derived species like *E. subornata, E. tomentosa *and *E. crispata *also feed on *Caulerpa *and retention of chloroplasts vary within these species. Feeding experiments with *Elysia crispata *by offering different algal speciesresulted in an increased activity after consumption of *Caulerpa verticillata*, whereas consumption of *Halimeda opuntia *did not contribute to photosynthetic activity [Grzymbowski & Wägele, unpublished data]. Although *E. timida *switches from hosts in the Cladophorales to algae in the Dasycladales during its life cycle, the chloroplasts inducing long-term photosynthetic activity apparently originate only from the dasycladalean *Acetabularia acetabulum *[41,42]. Newly hatched juveniles retain chloroplasts from *Cladophora dalmatica *[42], but quality of retention has never been investigated.

Long term retention in *Elysia chlorotica *is based on incorporation of chloroplasts from the heterokontophyte *Vaucheria*. Although *E. crispata *has been reported to consume both *Vaucheria *and a member of the Dasycladales, this species feeds principally on a variety of udoteacean green algae, and the origin of the chloroplasts responsible for long term photosynthetic activity is presently unknown [83]. The food of *Plakobranchus ocellatus *is unclear [65,72]; DNA barcoding is currently being used to identify the source of chloroplasts involved in long-term photosynthesis [Händeler et al., unpublished data].

### Measuring plastid functionality - methodological considerations

Use of PAM to quantify fluorescence of chlorophyll a in photosystem II reliably indicates whether chloroplasts are intact and functional within metazoan tissues. This method is non-invasive, can be easily applied in the field, and preserves the subjects for further genetic or histological analyses, including DNA barcoding studies to identify the source of sequestered chloroplasts. Direct assessment of chloroplast activity using a standardized method is an advance over prior work in which alternative methods were used to study different taxa, and plastid functionality was often assumed rather than demonstrated. For instance, Hinde and Smith [84] reported that chloroplast retention in *E. viridis *is functional for at least three months, based on a lone surviving slug (out of 39 specimens) which lost 96% of its body mass over the experimental period. Also, extrapolation of possible survival of slugs by analysing trendlines should be done with caution [45]. We do not adhere to previous classifications of photosynthetic activity into six [43] or even eight [45] different (artificial) levels, as the distinction between many of those proposed levels seems arbitrary according to our results in the present work.

We emphasize that although our data show trends for species, more detailed studies under standardized conditions are needed to resolve some remaining methodological issues. For instance, even when slugs are freshly collected from their food item, it is unknown how recently they consumed algal cytoplasm without direct observation of feeding; thus, only a minimum age can be determined for sequestered chloroplasts from field-caught specimens. In some species, photosynthetic activity may vary seasonally [85,41], geographically [85], or over the course of the life cycle [86,42]. Investigation of different ontogenetic states under starving conditions is thus warranted for species like *Elysia subornata *and *E. zuleicae*, to confirm whether the lack of chloroplast retention is limited to adult stages. Importantly, it remains to be determined whether chloroplasts of alternative host algae are retained for different periods of time by sacoglossans such as *Elysia crispata*, *E. viridis *and *E. timida *[87,25,83,86,88]. PAM studies on slugs that have fed on different algae are needed to rule out long-term retention of chloroplasts from a subset of potential hosts, before firm species-level conclusions can be drawn.

Additional work is also needed to assess how chloroplast retention is affected by abiotic factors such as light intensity and temperature [89-91]. Some sacoglossans exhibit behaviors that minimize exposure to high solar irradiance, which may prolong the activity of functional chloroplasts [91-93]. Parapodia, the lateral body flaps that cover the dorsum of species in the Plakobranchoidea, may have initially been an adaptation for shielding chloroplasts to reduce photosynthesis and, in consequence, production of toxic levels of intracellular oxygen. This resulted in an effective shielding of chloroplasts from destructive irradiance, hence prolonging the time of functional photosynthesis within the slugs. Finally slugs may even exert fine control over the light intensity to which chloroplasts are exposed by adjusting how far their parapodia are held open. *Plakobranchus ocellatus *was observed to roll its body and lie on the side to further reduce irradiance in bright sunlight [unpublished data of first and senior author]. Thus, anatomical and behavioral traits may together enhance the performance and survival of chloroplasts in different light regimes, and hence optimize the energy budget of slugs.

## Conclusion

Demonstrating that a key character promoted an adaptive radiation requires evidence of (1) a rapid burst of speciation, (2) the common ancestry of putative descendant species, and (3) the phenotype-environment correlation or trait utility, meaning the performance or fitness advantage of trait values in their corresponding environments [94]. Short-term retention of functional chloroplasts is a candidate key character that evolved once in the stemline of the Plakobranchoidea as has been shown here by comparative analyses. When not immediately digested, incorporated chloroplasts stay functional, hence provide fixed carbon, even if they degrade gradually over several days. This spares the slug from the energetic demands of feeding, when food availability decreases due to various ecological reasons (calcification of algae, migration in search for adequate mating partners, etc.) Kleptoplasty therefore likely contributed to the evolutionary success of the Plakobranchoidea. This success is evident both in the number of recognized species in the Plakobranchoidea, and in the ecological diversity of the group. Plakobranchoidea include more than 130 species and has five times more species than its putative sister taxon (family Hermaeidae and a part of the Polybranchiidae, see Figure 7) with about 25 species. Nevertheless, a robust estimate of the relative rate of speciation in the Plakobranchoidea will require additional data. Relationship of the Limapontioidea has to be clarified, and sister taxa relation of Plakobranchoidea needs confirmation. Furthermore, to apply statistical approaches as lineages-through-time plots a complete tree comprising all known species is necessary. More complete taxon sampling will be necessary to provide a robust estimate of relative rates of cladogenesis in different groups, permitting a statistical test of the key character hypothesis.

Ample studies demonstrate the trait utility of supplemental energy from kleptoplastids (see background). Evolution of parapodia typical only for Plakobranchoidea enables the slugs to keep chloroplasts alive and protect them against destructive irradiance. These structures even allow the selected exposure to different light intensities. Here we find anatomical as well as behavioral traits that optimize survival of chloroplasts in different light regimes and hence optimize energy budget and survival of slugs. We do not know at the moment how far this also affected switch to other host organisms and incorporation of "better" chloroplasts. It is evident that Plakobranchoidea, especially the species rich genus *Elysia *(> 80 species), have a broad range of food algae which they exploit (Figure 7). Future studies comprising the identification of the algal origin of incorporated functional chloroplasts will reveal any correlation between host organisms and photosynthetic activity of the slugs.

A second step in kleptoplastid evolution is the prolonged survival and photosynthetic activity of chloroplasts incorporated into the digestive cells of a slug. According to the phylogenetic hypothesis we present, this improvement in plastid retention occurred independently three times in *Elysia *and once in *Plakobranchus*. Although *Plakobranchus *is currently a monotypic genus, recent genetic studies indicate that it in fact comprises a clade of at least eight recently diverged cryptic species [P. Krug, A. Rodriguez, and C. Trowbridge, unpublished data] for which photosynthetic abilities have not been investigated so far.

Short-term retention may thus have been necessary to promote an adaptive radiation of the Plakobranchoidea. Other characters (e.g. host switch) or interactions between slug and host (e.g. usage of secondary metabolites) [95,96] may have additionally influenced speciation in Sacoglossa, and in particular adaptive radiation of the Plakobranchoidea (see also [97]).

After Jensen's [48,29] important first phylogenetic analysis and interpretations based on morphological data, we contribute here the next steps (molecular phylogeny and evolution of kleptoplasty). Nevertheless, only additional studies will clarify what trait combinations drove diversification within the Sacoglossa, and how the relationship between slugs and their host algae affected the evolution of this enigmatic opisthobranch taxon.

## Abbreviations

MRCA: most recent common ancestor; BF: Log-Bayes factor; PP: posterior probability.

## Competing interests

The authors declare that they have no competing interests.

## Authors' contributions

KH and PK generated sequence data and performed phylogenetic analyses. KH, YG and HW produced PAM measurements. KH analyzed PAM measurements. PK carried out character state reconstructions. KH, PK and HW wrote the manuscript. HW initiated and oversaw the project. All authors read and approved the final manuscript.

## Supplementary Material

Additional file 1 **Accession numbers of sequences and how they were concatenated**. Only 1^st ^and 2^nd ^position of *coxI *were used. id - sequence is from same specimen. Click here for file

Additional file 2 **Origin of specimens used for PAM measurements**. Place an date of collection are given, cipher in brackets depict number of specimens. Click here for file

Additional file 3 **Yield values for each specimen, mean values and standard deviation for species**. There is one table for each species. Full species name can be found in first line. All specimens of one species are listed with internal numbers. For single specimens collection date, date of first yield measurement, collection place and size are given in the table. Yield values are given for every specimen, mean value of yield values from every specimen per day of starvation after capture and standard deviation for one species are given. Mean values are plotted against days of starvation in figures 4 to 6. Standard deviation is plotted for species in figure 4 but not in figures 5 and 6 for better clearity. Click here for file
